# Items

**Published:** 1879-07

**Authors:** 


### Items.

List of Medical Officers of the U. S. Marine Hospital Service.—*Surgeon-General*—John O. Hamilton, Washington, D. C. *Surgeons*—E. Hebersmith, San Francisco, Cal.; C. N. Ellinwood, New York; P. H. Bailhache, Baltimore, Md.; John Vansant, Boston, Mass.; Wm. H. H. Hutton, New Orleans, La.; T. W. Miller, Chicago, Ill.; W. H. Long, Louisville, Ky.; Walter Wyman, St. Louis, Mo.; J. A. Brown, Detroit, Mich.; R. D. Murray, ordered to Norfolk, Va.; C. S. D. Fessenden, Portland, Me,; George Purviance, Pittsburg, Pa.; H. W. Sawtelle, Norfolk, Va.; E. J. Doering, Philadelphia, Pa. *Assistant Surgeons*—H. W. Austin, Key West, Fla.; Henry Smith, Galveston, Tex.; Jas. M. Gassaway, Pt. Townsend, W. T.; Geo. W. Stoner, Buffalo, N. Y.; J. C. Fisher, Cairo, Ill.; John Godfrey, Mobile, Ala.; F. H. Brown, Boston, Mass.; C. B. Goldsborough, Baltimore, Md.; Robt. White, on Rev. Str. "*Rush*"; H. M. Keyes, Cincinnati, O.; Geo. H. Stone, Savannah, Ga.; Proctor Thayer, Cleveland, O.; Fairfax Irwin, Chicago, Ill.; W. C. W. Glazier, N. Y.; Frank W. Mead, San Francisco, Cal.; Chas. L. Dana, (N. Y.) New York (temp'y); H. P. Cooke, (Va.) New Orleans, La.; H. R. Carter, (Md.) Boston, Mass. (temp'y); W. H. Heath, (Pa.) Washington, D. C. *Acting Assistant Surgeons*—J. H. O'Reilly, Evansville, Ind.; F. J. O'Connor, St. Louis, Mo.; F. D. Porter, Detroit, Mich.; A. C. Hamlin, Bangor, Me.; R. D. Bibber, Bath, Me.; Elmer Small, Belfast, Me.; K. H. Swett, Ellsworth, Me.; S. B. Hunter, Machias, Me.; Wm. A. Banks, Waldoboro', Me.; W. D. Stewart, Tuckerton, N. J.; H. G. Bates, New Berne, N. C.; Thos. F. Wood, Wilmington, N. C.; Jas. M. Allen, Milwaukee,

Wis.; K. S. Taft, Marquette, Mich.; Byron De Witt, Oswego, N. Y.; J. H. Van Deman, Chattanooga, Tenn.; Wm. M. Griffiths, Louisville, Ky. It will be seen in the above list, that Dr. E. J. Doering, late of Chicago, has been promoted to the rank of Surgeon. His many friends in this city will receive the news with pleasure.

CALIFORNIA'S LAST, BEST GIFTS.—In the May number of this journal, under the above title, an article appeared which was calculated to do injustice to one of our manufacturing houses, largely interested in the introduction of new remedies.

The article from which our excerpts were made originally appeared in the *Pacific Medical and Surgical Journal* for October, 1878. A subsequent number of this journal (January, 1879,) stated editorially, that "the former article was not intended to deny medicinal value to the plants in question, but simply to expose the deception of introducing preparations of old remedies under new names;" that is, in offering old remedies under coined names. But the editor effectually disposes of this charge by admitting that *Cascara Sagrada* is the common Spanish name for the drug in question, although he had previously stated that it was unknown to any botanist on the Pacific Coast. In fact, the original charges have been so modified that no further reply to them than the above statement seems necessary; still, to show how generally Dr. Gibbons' article has been condemned by the medical press, we add excerpts from editorials of some of the most prominent journals.

Physicians desirous of thoroughly investigating these charges will do well to send to Messrs. Parke, Davis & Co., of Detroit, for their pamphlet, containing all that has been published in relation to the matter.

"It shows prejudice, hence it is not scientific. We are sorry it is so, for the journal being indigenous to the same State as the plant, we expected real information."—*Chicago Pharmacist.*

"A great deal more has been made of the matter than was any occasion for. Of one of the remedies which is attacked, the cascara, we may say that it has now an excellent reputation in this locality."—*Louisville Med. News.*

" We are surprised that any enlightened member of the profession should object to any remedial agent because of its introduction by an eclectic."—*N. Y. Hospital Gazette.*

" But logic is logic, and failing to find anything objectionable in the remedies themselves, Dr. Gibbons opens his batteries on Dr. Bundy, the introducer of them. He proclaims the fact that Dr. Bundy is not a 'regular.' We must confess that we fail to perceive what Dr. B.'s 'regularity' or his lack of it has to do with the therapeutic properties of the drugs."—*Michigan Med. News.*

" We knew this firm (P. D. & Co.) was composed of shrewd business men, having it for their object to make an honest reputation and to obtain a fair return on their investment of brains, labor and capital. Hence we were satisfied that the originators of the damaging statements were mistaken."—*Detroit Lancet.*

" Those journals which made such haste to quote the first article from the *Pacific Journal* can hardly do less, in decency, than quote the second, and thus, as far as may be, make amends." —*Ohio Medical Recorder.*

" We were much astonished when we read Dr. Gibbons' article in the October number of the *Pacific.* * * * * Our own experience has taught that Cascara Sagrada, Grindelia Squarrosa and Yerba Santa are valuable remedies."—*St. Louis M. & S. Journal.*

"Although we know nothing of the orthodoxy of the professional status of Dr. Bundy, yet we claim to belong to that 'school of medicine' that hesitates not at the use of any drug, preparation or instrument that will benefit an afflicted fellow-being, no matter from what source it may have been derived, nor by whom introduced."—*Southern Practitioner.*

" It will be seen that Dr. Henry Gibbons here acknowledges that several of the plants in question are really valuable. This is all that the profession cares to know."—*Physician and Surgeon.*

